# Daily Contributors of Tinnitus Loudness and Distress: An Ecological Momentary Assessment Study

**DOI:** 10.3389/fnins.2022.883665

**Published:** 2022-07-05

**Authors:** Jorge Simoes, Jan Bulla, Patrick Neff, Rüdiger Pryss, Steven C. Marcrum, Berthold Langguth, Winfried Schlee

**Affiliations:** ^1^Department of Psychiatry and Psychotherapy, University of Regensburg, Regensburg, Germany; ^2^Department of Mathematics, University of Bergen, Bergen, Norway; ^3^Center for Neuroprosthetics, Institute of Bioengineering, École Polytechnique Fédérale de Lausanne, Lausanne, Switzerland; ^4^Department of Radiology and Medical Informatics, University of Geneva, Geneva, Switzerland; ^5^Department of Psychology, Center for Cognitive Neuroscience, University of Salzburg, Salzburg, Austria; ^6^Institute of Clinical Epidemiology and Biometry, University of Würzburg, Würzburg, Germany; ^7^Department of Otolaryngology, University Hospital Regensburg, Regensburg, Germany

**Keywords:** tinnitus, subtyping, ecological momentary assessment, distress, mood, mental health, loudness

## Abstract

**Background:**

Tinnitus is a heterogeneous condition which may be associated with moderate to severe disability, but the reasons why only a subset of individuals is burdened by the condition are not fully clear. Ecological momentary assessment (EMA) allows a better understanding of tinnitus by capturing the fluctuations of tinnitus symptoms, such as distress and loudness, and psychological processes, such as emotional arousal, overall stress, mood, and concentration and how these variables interact over time. Whether any of those variables have an influence over the next day, that is, whether any of these variables are auto- or cross-correlated, is still unanswered.

**Objectives:**

Assess whether behavioral and symptom-related data from tinnitus users from the TrackYourTinnitus (TYT) mobile app have an impact on tinnitus loudness and distress on subsequent days.

**Methods:**

Anonymized data was collected from 278 users of the iOS or Android TYT apps between 2014 and 2020. Tinnitus-related distress, tinnitus loudness, concentration level, mood, emotional arousal, and overall stress level were assessed using either a slider or the Wong-Baker Pain FACES scale *via* a daily survey. Three modeling strategies were used to investigate whether tinnitus loudness and distress are affected by previous days symptoms or psychological processes: auto- and cross correlations, regressions with elastic net regularization, and subgrouping within group iterative multiple model estimation (S-GIMME).

**Results:**

No autocorrelation or cross-correlation was observed at the group level between the variables assessed. However, application of the regression models with elastic net regularization identified individualized predictors of tinnitus loudness and distress for most participants, with the models including contemporaneous and lagged information from the previous day. S-GIMME corroborated these findings by identifying individualized predictors of tinnitus loudness and distress from the previous day.

**Discussion:**

We showed that tinnitus loudness and tinnitus distress are affected by the contemporaneous and lagged dynamics of behavioral and emotional processes measured through EMA. These effects were seen at the group, and individual levels. The relevance EMA and the implications of the insights derived from it for tinnitus care are discussed, especially considering current trends toward the individualization of tinnitus care.

## Introduction

Tinnitus is a condition in which phantom sounds are perceived without a corresponding external stimulus. Those sounds usually take the form of ringing, hissing, or buzzing, but other less common types of perception have also been reported (Baguley et al., 2013; Langguth et al., 2013). The underlying causes of tinnitus are not fully clear, but auditory pathway deafferentation is commonly recognized as key factor in the etiology of tinnitus (Shore et al., 2016). Although tinnitus is generally a benign condition, its bothersome manifestation, which is estimated to affect 1% of the population (Biswas and Hall, 2020), can be both debilitating (Cima, 2018) and costly (Maes et al., 2013). Tinnitus can be subdivided into two categories: acute and chronic (de Ridder et al., 2021). The former category describes the rather common phenomenon of phantom sounds being perceived for several seconds or minutes after insult to the auditory system (e.g., listening to loud music); whereas the latter category refers to uninterrupted perception of the phantom sound for at least 3 months. In its chronic presentation, tinnitus rarely resolves entirely (Tunkel et al., 2014; Simões et al., 2021).

Available treatments are not effective at suppressing the chronic phantom perception for most sufferers. As a result, most treatment strategies seek to reduce tinnitus-related distress (Tunkel et al., 2014). There is a growing consensus that tinnitus is a heterogeneous condition and that this characteristic may significantly impact both how the condition is experienced by patients and the efficacy of treatments (Simoes et al., 2019; Kleinjung and Langguth, 2020). Factors demonstrated to affect treatment outcomes include sociodemographics, personality, and tinnitus characteristics, though many others are currently being investigated (Genitsaridi et al., 2020). For example, the heritability of tinnitus differs depending on its laterality (i.e., whether sounds are perceived in one or both ears) and the patient's gender (Maas et al., 2017). Furthermore, evidence suggests that personality traits may explain the response to online cognitive behavior therapy treatment (Kleinstäuber et al., 2018), but not acoustic stimulation (Hafner et al., 2020). It is unclear which factors are related to tinnitus-related disability, as the psychoacoustic properties of tinnitus (e.g., laterality, loudness, pitch, type of perceived sound) do not fully explain the tinnitus distress (Cederroth et al., 2019; Kleinjung and Langguth, 2020). Therefore, it is of great clinical interest to understand which factors are associated with distress, especially at the individual level.

The advent of minimally intrusive longitudinal sampling methods has allowed researchers and clinicians to develop predictive models at the individual level, while at the same time maximizing the ecological validity of assessments (Wright and Woods, 2020). Not surprisingly, there is growing interest in using ecological momentary assessment (EMA) in the fields of psychology and psychopathology in general (Wright and Woods, 2020), as it provides clinicians and researchers a tool to investigate the interplay between emotions, symptoms, and behaviors, while considering contextual associations (e.g., how mental phenomena co-occur and interact over time) in naturalistic settings (Shiffman et al., 2008). In the context of tinnitus, Probst et al. (2016), modeled patterns of daily fluctuation of tinnitus characteristics and identified a mediatorial role of tinnitus loudness on stress, while Pryss et al. used EMA to establish that patients often have recollection bias regarding tinnitus fluctuations throughout the day (Pryss et al., 2019). However, most of the studies in the tinnitus field using EMA have focused on group-level analysis. A recurring clinical topic is how uniquely tinnitus is experienced by patients, with different triggers leading to higher tinnitus distress and/or loudness in certain patients, but not others. Identification of factors that could lead to personalized interventions is of great clinical utility.

Therefore, the objective of this study was to apply EMA 206 methods to identify factors (see Table 2) that could be associated with tinnitus loudness and distress at both the individual and group levels. Furthermore, data collected used EMA sampling were used to identify whether such factors influence loudness and/or distress on a subsequent day. To achieve these objectives, we first investigated whether states such as tinnitus loudness, distress, and mood impact autocorrelates or cross-correlates throughout subsequent days at the group level. Second, we modeled data at the individual level using linear regressions with elastic net regularization for each unique time series with tinnitus distress and tinnitus loudness as dependent variables. Third, we used subgrouping within group iterative multiple model estimation (S-GIMME) to obtain unique models for each participant on contemporaneous and lagged effects between the variables collected with EMA.

## Methods

### Data Preparation

The data analyzed in this study were collected between 2014 and 2020 from the “TrackYourTinnitus” (TYT) app, which is freely available on both Android and iOS mobile devices (available as TrackYourTinnitus). After registration, participants complete an 8-question survey with questions related to their current perception of tinnitus and mood. Users are free to use the app for an indeterminate time and without frequency restrictions. An overview of the variables included in this study is available in **Table 2**. Two variables were excluded from this study: “do you perceive your tinnitus right now?” and “do you feel irritable right now?”, as those questions were binary and continuous variables were necessary to obtain auto- and cross-correlations. The remaining variables were rated either with a slider (questions 2, 3, 6, 7) or using a variation of the Wong-Baker pain FACES scale [WBS, questions 4–5, (Garra et al., 2013)]. Variables measured with a slider ranged from 0 to 100, and variables measured with the WBS could be answered with a 9-point scale, with each figure representing different levels of discomfort. Both types of variables were treated as continuous (Rhemtulla et al., 2012). Informed consent was obtained from users to have their data anonymously used for scientific purposes. The study was approved by the Ethics Committee of the Faculty of Medicine of the University of Regensburg (Study approval number 15-101-0204).

During registration, users were asked to fill-in two questionnaires: the Mini-Tinnitus Questionnaire (Hiller and Goebel, 2004) and the Tinnitus Sample Case History Questionnaire, TSCHQ (Langguth et al., 2007). In addition, users responded to a question about their worst tinnitus-related symptom. Mini-TQ is commonly used in clinical trials and ambulatory assessment as a screening tool for tinnitus-related distress. The questionnaire possesses good psychometric properties (correlation > 0.9 with the original 52-item Tinnitus Questionnaire, test-retest reliability of 0.89, and Cronbach's alpha of 0.9) and consists of 12 questions. The second questionnaire is part of an international effort to standardize data collection and reporting in tinnitus research and is also a standard screening tool. The TSCHQ consists of 34 questions related to tinnitus characteristics (e.g., the type of perceived sounds, duration of tinnitus, subjective loudness), life history (e.g., whether family members also suffer from tinnitus), and common comorbidities (e.g., headaches, insomnia, hearing aids). Both questionnaires were used for the description of the sample.

Regarding the usage of app, users could set push notifications to on or off and were allowed to report their status at any time and as often as they wanted. Only time series datasets with at least 10 days of consecutive sampling were included in the analysis as a compromise between having enough data to model the data and not excluding too many data-points due to its length, as longer streaks of sequential observations become increasingly rarer (See Supplementary Figure 1). If the same user had two sequences of observations that lasted at least 10 days, those two sequences were analyzed independently. The results reported in this article were obtained by selecting the first observation of each day. Missing values from a given sequence were imputed using the “aregImpute” function from the Hmisc package with default settings in R.

### Statistical Analysis

Different statistical techniques were used to describe the relation between the variables collected with EMA, both at the group and individual levels. Autocorrelations and cross-correlations were used to obtain statistical associations at the group level, whereas linear regressions with elastic net regularization and unified structural equation modeling were used to obtain individualized models. These methods are described in the following.

### Auto- and Cross-Correlation

Autocorrelation can be described as the correlation of a variable with itself at different time lags (Beal and Weiss, 2003). Likewise, cross-correlations measure the correlation between two variables (e.g., tinnitus loudness and distress) at different timepoints. This way, we could estimate whether any of the six variables shown in **Table 2** are related with themselves (e.g., autocorrelation) or with one another (e.g., cross-correlation) at subsequent days at the group level.

### Elastic Net Regularization

Elastic net regularization is an increasingly popular method intended to account for datasets with large numbers of predictors, especially when those may be correlated. Elastic net shrinks the coefficients of correlated predictors and performs feature selection by setting some of them to 0 (Zou and Hastie, 2005). This property of the model was used to build individualized models for each sequence of observations to predict both tinnitus loudness and tinnitus distress.

All variables in the regression were modeled as being linearly related to the outcome measures. A 10-fold cross-validation was computed with the default settings using the cv.glmnet function from the glmnet package. The final model was selected based on the lambda one standard error from the minimum for parsimonious results (Zou and Hastie, 2005).

### Unified Structural Equation Modeling

Unified Structural Equation Modeling (uSEM) combines structural equation modeling and vector autoregression and can be used to extract autoregressive and cross-lagged effects from time series. As a result, uSEM has been used widely in psychological and medical science to estimate contemporaneous and lagged effects neuropsychological phenomena such as brain activity recorded from functional magnetic resonance imaging and behavioral/emotional fluctuations recorded by EMA. Additionally, uSEM allows for estimates to be calculated at the individual, subgroup, and group levels. The validity and reliability of this method in obtaining individualized estimates from intensive longitudinal data sampling have been previously discussed (Wright and Woods, 2020) and explored both with simulated and empirical data (Lane et al., 2019). For this analysis, uSEM was estimated using the gimmeSEM function with the subgrouping option from the gimme package (Gates et al., 2016).

All analyses were performed in R (version 4.0.1, R Core Team, 2018). Mathematical descriptions of auto- and cross-correlations, as well as elastic net regularization and uSEM are presented in the Supplementary Materials. Auto- and cross-correlations were conducted with an internal script adapted by JB from the functions “acf” and “ccf” available in R. The adapted functions calculate weighted average auto- and cross correlations and therefore were applied to multiple sequences of observations.

## Results

Table 1 summarizes the demographics of the samples used for both analyses. Of the original dataset, 57% of the data were excluded from the analysis as the data were not obtained from that sequence for at least 10 uninterrupted days (see Supplementary Figure 1). Thus, the sample of study 1 consisted of 488 unique sequences from 278 users. Following the guidelines of the authors of the S-GIMME package, only sequences with at least 60 days of uninterrupted usage were included in the second analysis (Lane et al., 2019). Thus, the sample consisted of 32 sequences from 32 unique users. Table 2 shows how the EMA questions were formulated (translated into English from German), with their abbreviations. Two questions, namely questions 1 and 8, were excluded from the analysis as they were dichotomous and continuous variables were necessary for computing auto- and cross-correlations.

**Table 1:** Sample demographics collected *via* the TSCHQ during registration in the APP.

	**Analysis 1** **(*N* = 278)**	**Analysis 2** **(*N* = 32)**
**Age**		
Mean (SD)	55.3 (12.8)	55.4 (7.09)
Median [Min, Max]	56 [23.00, 83.0]	54.0 [40.0, 65.0]
Missing	160 (57.5)	18 (56.2%)
**Tinnitus onset (months)**		
Mean (SD)	18.7 (13.4)	17.6 (11.9)
Median [Min, Max]	13.4 [2.50, 69.3]	15.0 [6.00, 47.0]
Missing	89 (32%)	17 (53.1%)
**Gender**		
Female	201 (72.3%)	28 (87.5%)
Male	73 (26.3%)	4 (12.5%)
Missing	4 (1.4%)	-
**Subjective tinnitus loudness**		
Mean (SD)	49.1 (28.5)	49.5 (29.8)
Median [Min, Max]	50.0 [0, 100]	52.0 [0.5, 94.0]
Missing	56 (20.1%)	7 (21.9%)
**Type of perceived sound**		
Crickets	31 (11.2%)	3 (9.4%)
Noise	54 (19.4%)	8 (25.0%)
Other	15 (5.4%)	1 (3.1%)
Tone	169 (60.8%)	19 (59.4%)
Missing	9 (3.2%)	1 (3.1%)
**Initial perception**		
Abrupt	133 (47.8%)	19 (59.4%)
Gradual	141 (50.7%)	13 (40.6%)
Missing	4 (1.4%)	–
**Mini-TQ**		
Mean (SD)	13.7 (5.85)	13.9 (4.90)
Median [Min, Max]	14.0 [0, 24.0]	12.5 [3.00, 23.0]
Missing	5 (1.8%)	–

*Sample 1 provided sequences with at least 10 days of uninterrupted usage used for auto- and cross-correlation as well as for elastic net regressions. Sample 2 provided sequences with at least 60 days of uninterrupted usage used for uSEM*.

**Table 2:** TYT questions included in the study.

**Item**	**Translation of the German question**	**Abbreviations**
Question 2	How loud is your tinnitus right now?	LO
Question 3	How stressful is your tinnitus right now?	TD
Question 4	How is your mood right now?	MO
Question 5	How is your emotional arousal right now?	AR
Question 6	Do you feel stressed right now?	ST
Question 7	How much are you concentrating on the things you are doing right now?	CO

*Question 1 (“Do you perceive your tinnitus right now?”) and question 8 (“Do you feel irritable right now?”) were excluded as their answers were dichotomous*.

**Figure 1:**
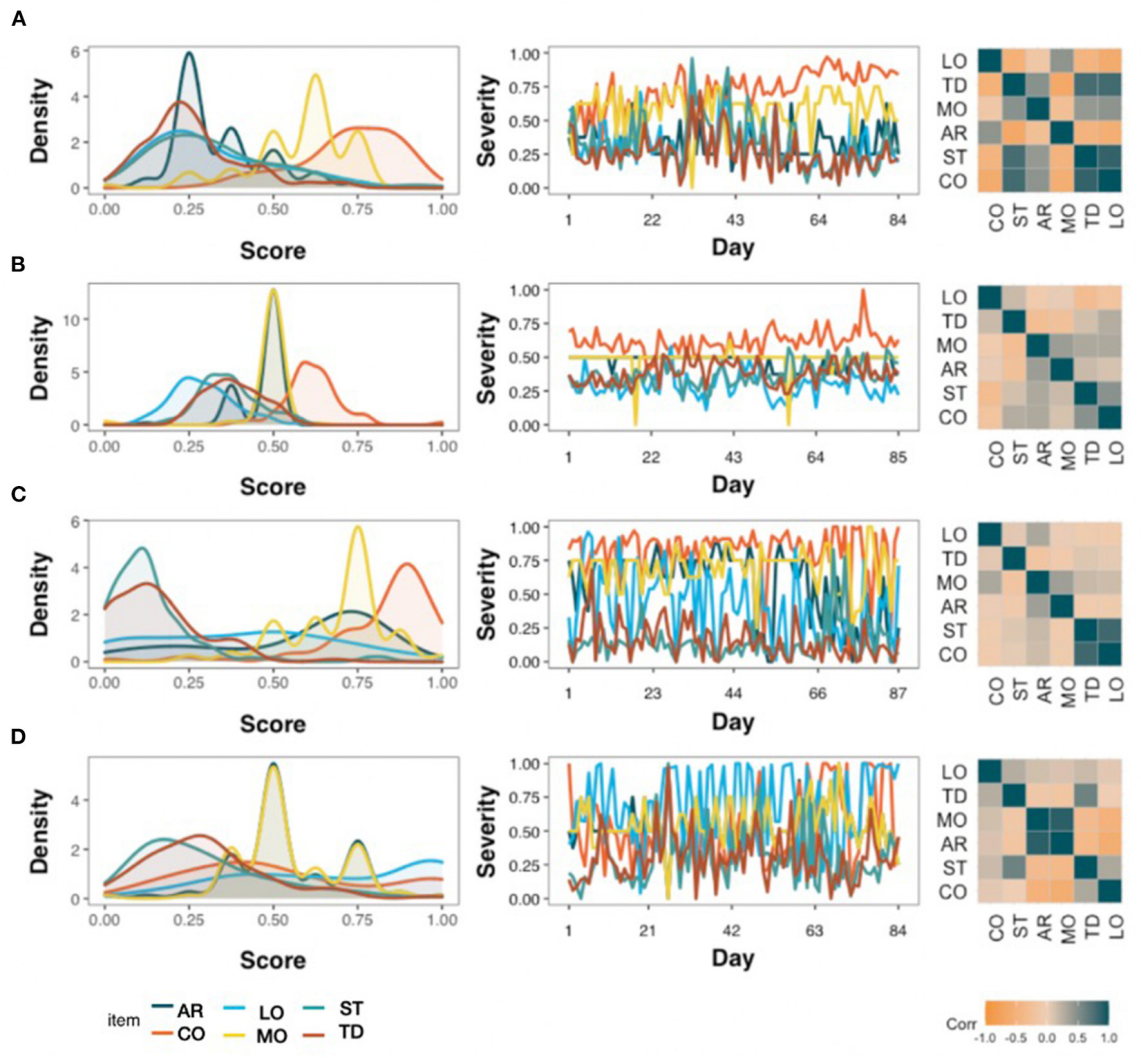
Variability between four TYT users, each represented in one row **(A–D)** who completed around 85 days of continuous EMA. Density plots (left), time series (middle) and correlation heat maps (right) highlight how emotional arousal (AR), concentration (CO), tinnitus loudness (LO), mood (MO), stress (ST), and tinnitus distress (TD) interact with each other.

Figure 1 illustrates how uniquely tinnitus is uniquely experienced by four arbitrarily selected TYT users. The left column of the figure shows the mean values and their dispersion with density plots; the middle column shows the fluctuation of those variables through time with time series plots; the right column depicts the contemporaneous relation between variables with correlational heat maps. Overall, these four examples highlight how symptoms may be burdensome, how they fluctuate over time, and how they interact with each other.

### Auto and/or Cross-Correlations Between Tinnitus Loudness, Distress, and Variables Related to Mood

**Figure 2:**
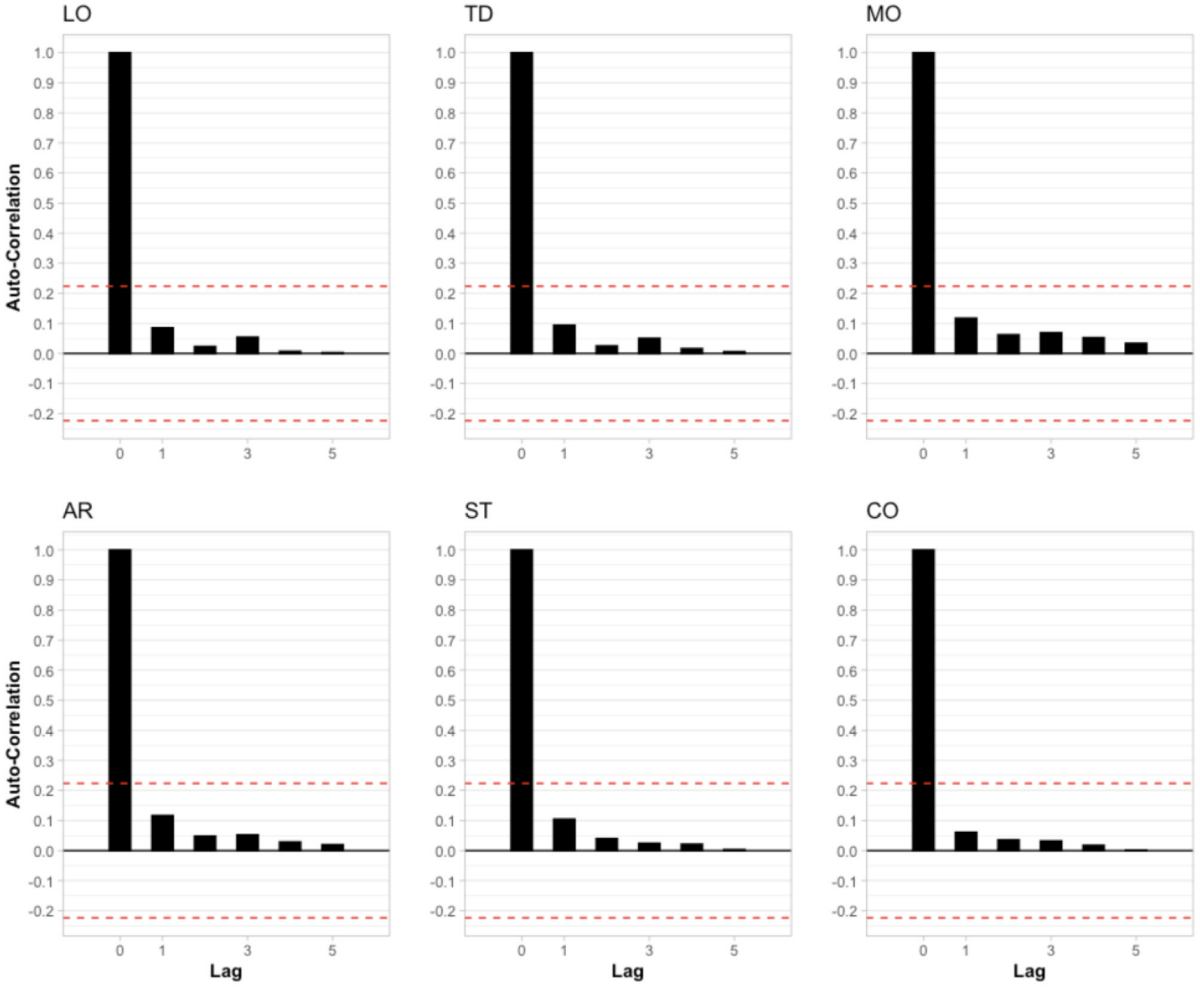
Autocorrelation of the six variables included in the analysis. Each lag represents a different day of usage. Red dashed lines represent the 95% confidence interval. LO, Loudness; TD, Tinnitus Distress; MO, Mood; AR, Arousal; ST, Stress; CO, Concentration.

**Figure 3:**
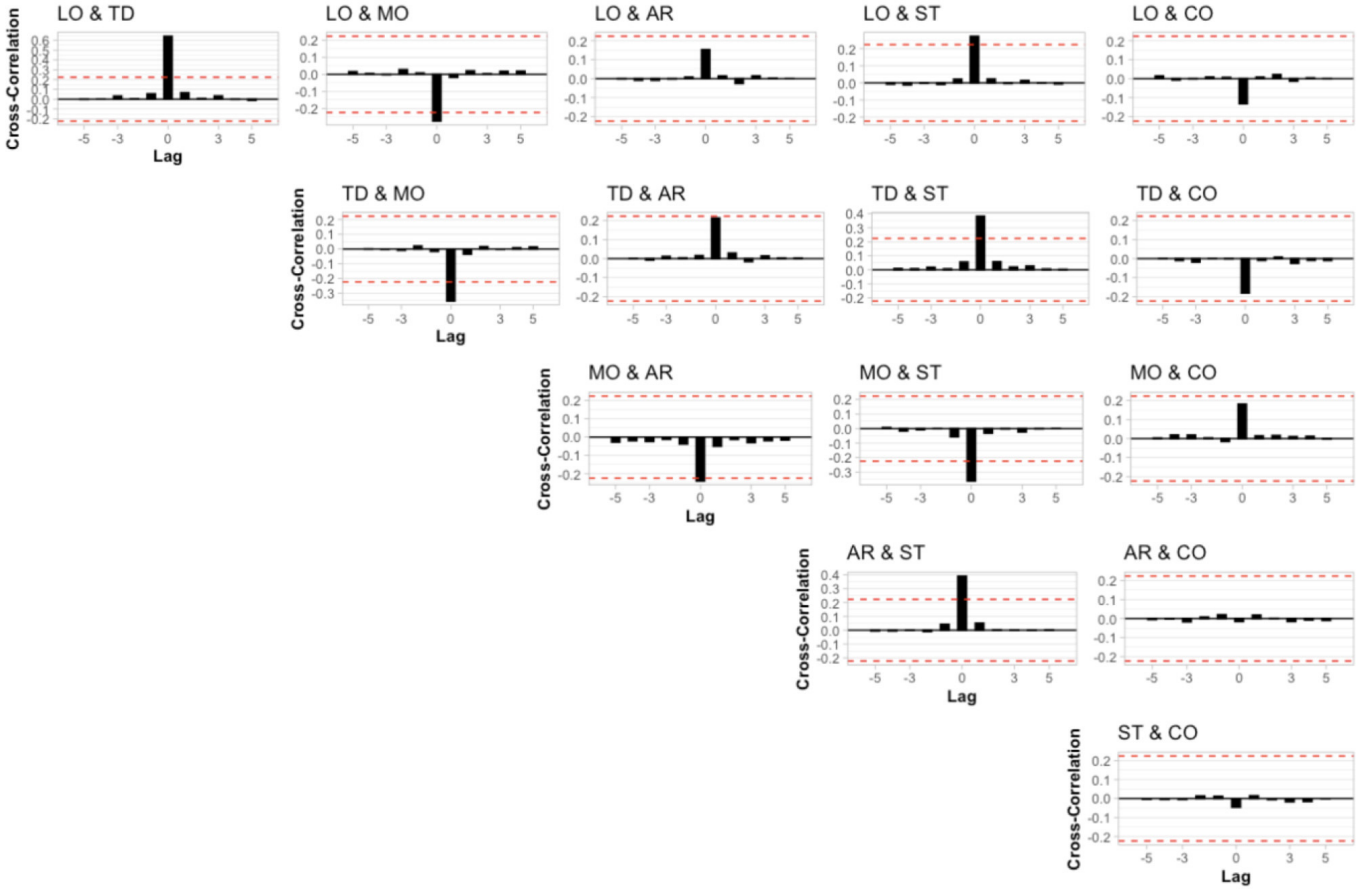
Cross-correlation of the potential combinations of the six variables included in the analysis. Each lag represents a different day of usage. Red dashed lines represent the 95% confidence interval. LO, Loudness; TD, Tinnitus Distress; MO, Mood; AR, Arousal; ST, Stress; CO, Concentration.

First, we investigated whether the six variables were autocorrelated (see Figure 2). None of the lagged variables was outside the 95% confidence interval (red dashed lines), suggesting that there was no autocorrelation. Autocorrelations in lag 0 were always 1, since the nominators and denominators were identical in those cases (see Supplementary Equation 1). Next, we investigated whether there was cross-correlation between the variables (see Figure 3). Like the previous results, no correlation at lags > 0 was observed. However, corroborating previous findings, we observed contemporaneous correlations (i.e., at lag 0) between loudness & tinnitus distress, tinnitus distress & mood, tinnitus loudness & stress, tinnitus distress & stress, mood & emotional arousal, mood & stress, and arousal & stress.

### Individualized Models With Elastic Net Regularization

**Figure 4:**
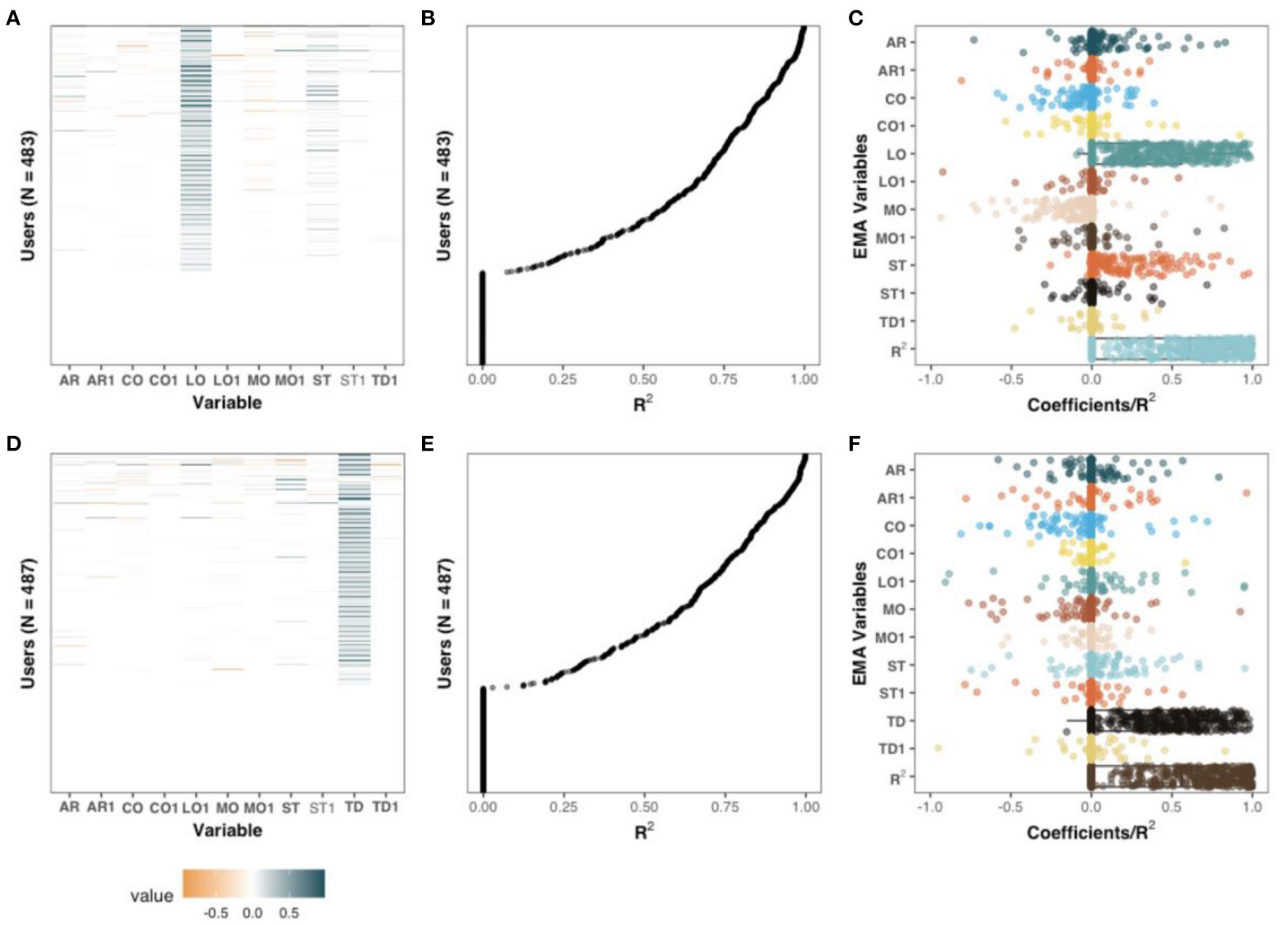
Coefficients of penalized regression models with TD **(A–C)** and LO **(D–F)** as dependent variables. Sequences in which the dependent variable had no variability were excluded from this analysis. The coefficients and *R*^2^ of the individualized models on the *Y*-axis of **(A,B)** and **(D,E)** are aligned. Left column: standardized coefficients uniquely estimated for each sequence. Middle column: the amount of variance explained, measured with *R*^2^, for each unique sequence. Right column: The adjusted coefficients of **(A,D)**, and *R*^2^, of **(B,E)** are presented with box and dot plots.

Next, we investigated whether elastic net regressions could be used for individualized inference about tinnitus loudness (see Figures 4A–C) and tinnitus distress (see Figures 4D–F). For this analysis, contemporaneous variables and lagged variables from the previous days (acronyms ending with “1” in Figures 4A,C,D,F) were used as independent variables in regression setups. For 27% and 31% of the sample, no predictors of loudness and tinnitus distress were found (see Figures 4A,D, respectively). For the remaining sample, the *R*^2^ for each time series varied considerably (see Figures 4B,E). Figures 4C,F summarize these findings with box plots. Although certain variables were almost only positively associated with the outcome measures (e.g., stress), other variables presented both positive and negative valence throughout the sample (e.g., concentration and emotional arousal).

Based on the model coefficients, positive associations were observed between tinnitus loudness and tinnitus distress among 314 users (65%) at the contemporaneous level and 17 (3.52%) users at the lagged level. stress was positively associated with tinnitus distress for 133 users (27.5%) and mood was negatively associated with tinnitus distress in 105 (21.7) cases. The associations between tinnitus distress and the remaining variables, positive or negative, were present only among a fraction of users (0.6–11.4%). Apart from the association between loudness and tinnitus distress, few associations with tinnitus loudness (0.2–11%) were observed. Stress was positively associated with tinnitus loudness for 53 (11%) of the users, and mood was negatively associated with 50 users (10.4%).

### Idiographic Modeling With S-GIMME

Only time series with at least 60 sequential observations were included in this analysis, yielding a sample size of 32 unique users. The models converged in all cases and a good fit was observed: [average: chi-square (44, *N* = 32) = 65.56, *p* = 0.12, comparative fit index = 0.98, root-mean square error of approximation = 0.06, non-normed fit index = 0.96, standardized root mean residual = 0.06]. Individual model fits are available as Supplemental Material.

**Figure 5:**
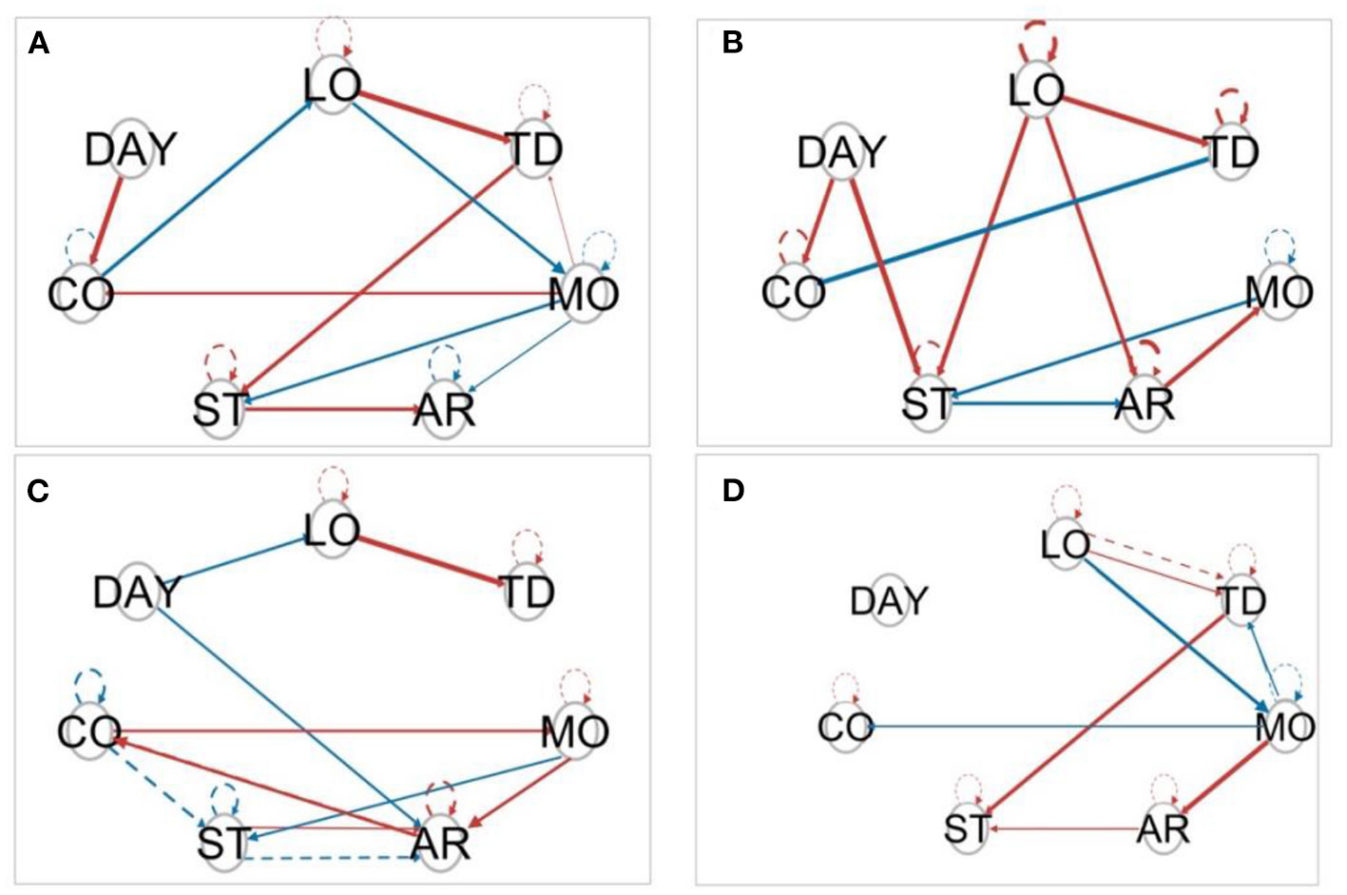
Model estimated by S-GIMME by the same four **(A–D)** individuals from Figure 1. Solid lines represent a contemporaneous effect (lag = 0), and dashed lines represent lagged effects (lag = 1). The circles represent the variables included in the study. Arrows indicate the direction of the relationship. Red arrows indicate positive regression paths and blue lines indicate negative regression paths.

Figure 5 shows the paths for the four individuals from Figure 1. The variable “Day”, that is, the position in the time series was encoded as an exogenous variable, meaning that the variable could predict any other variables, but not the other way around. Both contemporaneous (solid) and lagged (dashed) paths were obtained in all cases, including the four highlighted cases in Figure 5. Although some paths were shared across subjects, e.g., the effect of tinnitus distress on loudness, other dynamics were idiographic (e.g., whereas stress had a positive contemporaneous effect on tinnitus distress for user shown in Figure 5A, the relationship was inverted for the user shown in Figure 5D, and no relationship was seen for the other two users).

### Description of Subgroups Identified by S-GIMME

**Figure 6:**
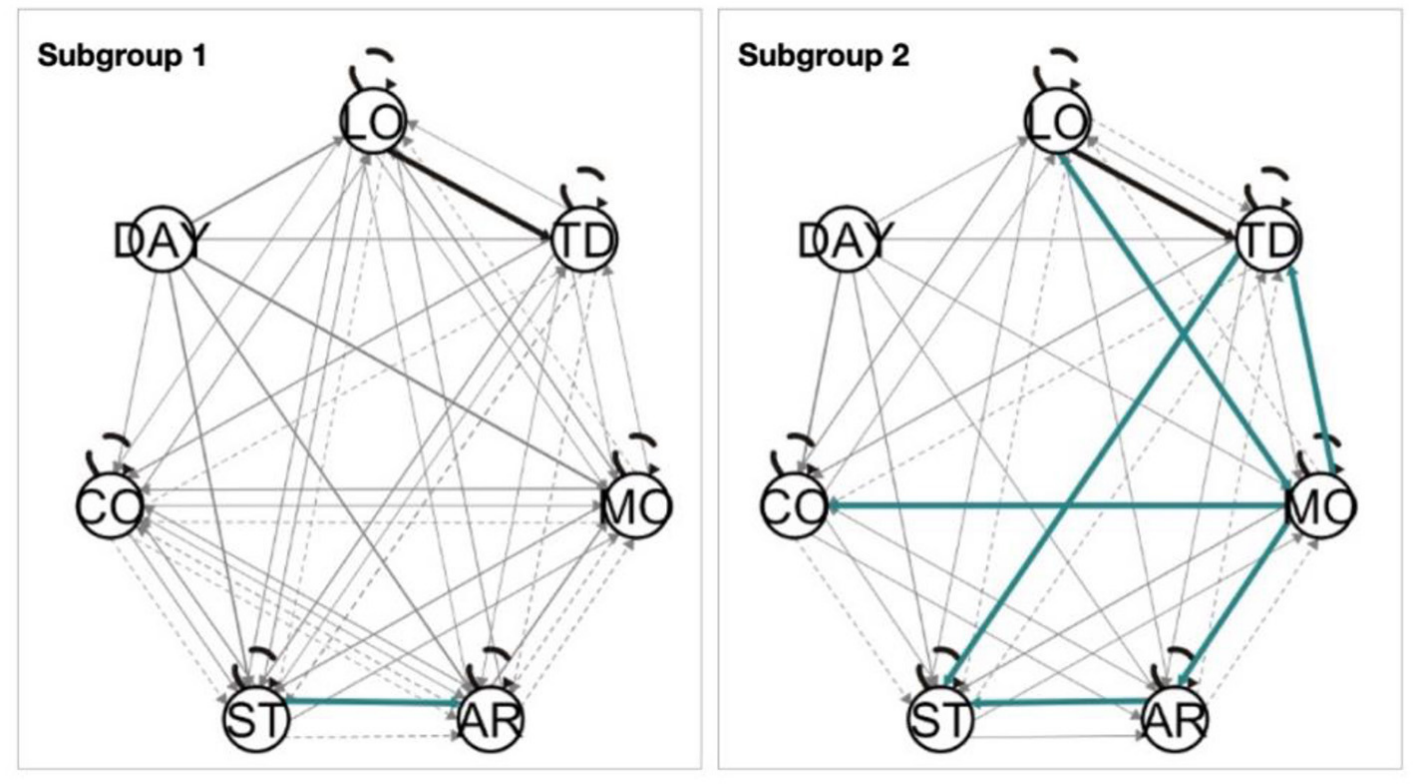
Two subgroups were identified by S-GIMME (left: *N* = 19, right: *N* = 13). The black lines represent the paths present at the group level (e.g., paths shared by both subgroups), the green lines represent the paths present at the subgroup level, and the gray lines represent the paths at the individual level. Solid lines represent contemporaneous effects, and dashed lines represent lagged effects. Arrow heads indicate the direction of the relationship.

S-GIMME identified two subgroups based on the dynamic similarities between variables collected through EMA. Group 1 consisted of 19 individuals, while group 2 consisted of 13 individuals. No differences were observed between the subgroups in terms of mean EMA scores, sociodemographics, or tinnitus characteristics (see Table 3). The paths obtained from S-GIMME for each subgroup are shown in Figure 6. Interestingly, most of the lagged relations were observed in subgroup 1 (i.e., dashed lines), whereas subgroup 2 consisted mainly of contemporaneous (i.e., solid lines), suggesting that S-GIMME could distinguish users whose tinnitus is mostly modulated from contemporaneous effects from users whose tinnitus is modulated by dynamics from the previous day. The green paths shown in Figure 6 highlight effects specific to all members of a subgroup. Of interest, most of the effects specific for subgroup 2 originated from either tinnitus distress or loudness. These findings suggest that S-GIMME could not only distinguish users with contemporaneous or lagged dynamics, but also users whose tinnitus distress and tinnitus loudness were associated with another, and with emotional arousal, stress, and mood (see Figure 6, right).

**Table 3:** Description of the 32 users included in the analysis with S-GIMME.

	**Subgroup 1** **(*N* = 19)**	**Subgroup 2** **(*N* = 13)**	***p*** **-value**
**Age**			0.55
Mean (SD)	50.9 (8.8)	52.8 (10.1)	
Median [Min, Max]	55.1 [35.0, 63.8]	51.6 [31.5, 67.5]	
Missing	1 (5.3%)	1 (7.7%)	
**Tinnitus onset (months)**			0.55
Mean (SD)	140 (158.0)	86.2 (82.0)	
Median [Min, Max]	66.1 [2.4, 529]	43.2 [6.3, 243]	
Missing	1 (5.3%)	1 (7.7%)	
**Mean LO**			0.71
Mean (SD)	0.4 (0.3)	0.367 (0.197)	
Median [Min, Max]	0.4 [0.1, 0.9]	0.3 [0.1, 0.8]	
**Mean TD**			0.84
Mean (SD)	0.3 (0.2)	0.3 (0.1)	
Median [Min, Max]	0.3 [0.1, 0.9]	0.3 [0.1, 0.4]	
**Mean AR**			0.37
Mean (SD)	0.2 (0.2)	0.2 (0.1)	
Median [Min, Max]	0.2 [0.01, 0.6]	0.2 [0.1, 0.5]	
**Mean CO**			0.56
Mean (SD)	0.5 (0.3)	0.6 (0.2)	
Median [Min, Max]	0.5 [0.2, 0.9]	0.7 [0.1, 0.8]	
**Mean ST**			0.28
Mean (SD)	0.2 (0.1)	0.3 (0.1)	
Median [Min, Max]	0.2 [0.1, 0.5]	0.3 [0.1, 0.5]	
**Gender**			0.89
Male	3 (15.8%)	1 (7.7%)	
Female	16 (84.2%)	12 (92.3%)	
**Subjective tinnitus loudness**			0.19
Mean (SD)	47.2 (28.3)	53.7 (33.7)	
Median [Min, Max]	46.0 [0.7, 84.0]	62.0 [0.5, 94.0]	
Missing	3 (15.8%)	4 (30.8%)	
**Initial perception**			0.87
Abrupt	12 (63.2%)	7 (53.8%)	
Gradual	7 (36.8%)	6 (46.2%)	
**Mini-TQ**			0.83
Mean (SD)	13.7 (5.4)	14.2 (4.3)	
Median [Min, Max]	12.0 [3.0, 23.0]	13.0 [8.0, 21.0]	

*P-values were obtained from the Kruskal-Wallis test if the response variable was continuous or from Chi-square tests if the response variable was categorical. No corrections were made for multiple comparisons*.

## Discussion

In this study, we identified individuals whose tinnitus loudness and distress are affected by behavioral and emotional processes of the same and of the previous day. We started by showing that at the group level, no evidence of autocorrelation or cross-correlation could be observed between the six EMA variables. However, modeling data at the individual level revealed that tinnitus loudness and distress were auto- and cross-lagged from one day to another for several individuals (see Figures 3–5). Lastly, we used S-GIMME to explore the unique interplay of these variables, effectively modeling how heterogeneously tinnitus manifests itself over time.

Our results provide a template for how to model tinnitus loudness and tinnitus distress at the individual level (see Figures 3–5). The interaction between these variables constitutes a complex mosaic of how tinnitus is experienced; Although the uniqueness of experiencing tinnitus has been widely acknowledged (Tunkel et al., 2014; Elgoyhen et al., 2015; Cederroth et al., 2019), empirical studies that demonstrate this complex relationship were lacking. Furthermore, using S-GIMME, we were able to incorporate the effect of time into the models, a critical component of experiencing tinnitus (Probst et al., 2017) often neglected in empirical studies due to the challenges of conducting multiple samplings in the traditional ambulatory setting.

Corroborating previous findings, we showed a positive association between stress, tinnitus loudness, and tinnitus distress (Scott et al., 2009; Probst et al., 2016). Interestingly, we also observed that emotional arousal, mood and concentration had ambivalent associations with both loudness and distress (see Figures 4C,F). Such associations could explain why tinnitus is uniquely experienced, and future research could further investigate factors associated with tinnitus distress and loudness, such as behavior, emotional and cognitive dynamics samples from EMA. Tinnitus is known to have potential negative consequences on cognition (Andersson and McKenna, 2009; Mohamad et al., 2016; Neff et al., 2021), but this is the first time a positive association between concentration and tinnitus distress/loudness has been shown. Future studies should further investigate this seemingly paradoxical relationship, especially considering that concentration problems are one of the core domains that tinnitus patients would like to have as outcome measures from clinical interventions (Hall et al., 2018, 2019). For example, this positive association could be related to compensatory mechanisms.

Other core domains include the ability to ignore the phantom perception, its intrusiveness, the sense of control over one's body, quality of sleep, and negative thoughts and beliefs. The first three domains could be investigated by adapting questions from the Tinnitus Functional Index, a widely used, validated questionnaire that captures different dimensions of tinnitus distress (Meikle et al., 2012). Negative thoughts could be investigated by adapting the positive and negative affect schedule (Watson et al., 1988), similar to other studies in the field of psychopathology using EMA (Wright and Woods, 2020; Heller et al., 2021). Sleep problems remain one of the main complaints of clinical tinnitus patients (Tunkel et al., 2014; Asnis et al., 2018; Lu et al., 2020; Inagaki et al., 2021; Richter et al., 2021), but no study to date investigated its effect using an EMA design, despite the evidence of its relevance on other chronic, disabling conditions (Short et al., 2017).

Our work suggests that only a subset of patients experience lagged effects between the variables sampled through EMA (see Figures 4–6). This finding could be leveraged to deliver personalized interventions through mobile apps, especially among users where tinnitus distress and loudness and auto or cross-correlated. For example, the results suggest that patients with tinnitus may benefit from just-in-time adaptive intervention (JITAI) (Nahum-Shani et al., 2016; Wang and Miller, 2019). JITAI uses mobile sensing to deliver customized interventions based on unique fluctuations recorded by the EMA. Such a system has been used in several fields, including physical health, addiction, and mental care research, but not in tinnitus (Wang and Miller, 2019). For example, customized interventions could be delivered to patients whose tinnitus loudness and/or distress are auto- or cross-correlated over days: Once the algorithm anticipates a potential spike in tinnitus loudness or stress on the next day, an intervention such as psychoeducation tips for coping with tinnitus through push notifications (Unnikrishnan et al., 2020), sound therapy (Tyler et al., 2018; Kutyba et al., 2019, 2021), online delivered cognitive behavior techniques (Weise et al., 2016; Kleinstäuber et al., 2018; Andersson et al., 2019), or meditation techniques (Fitzgerald et al., 2021) could be activated. Future studies could evaluate whether these results are replicated with different sampling frequencies. For example, an EMA every 8 h has been shown to be well tolerated in clinical settings (Shiffman et al., 2008) and would considerably reduce the duration of the study, and potentially increase the adherence to using the APP (Schleicher et al., 2020).

Methodological limitations should be considered when interpreting these results. For example, missing values continue to constitute a significant challenge for researchers, including those using EMA. We implemented a popular and robust method for data imputation; however, empirical evidence that this is the optimal method for imputing data from EMA is not available. A recent study investigated the causes of discontinuation of TYT use (Schleicher et al., 2020), but no clear predictors of adherence to app use were found. Furthermore, results suspected of bias cannot be easily discarded, as only a fraction of users used the app for more than 10 consecutive days (Supplemental Material). Therefore, it is unlikely that our sample is representative of the whole population, and caution should be exercised when interpreting the results and its generalizability. The possibility of low stability from the coefficients obtained from elastic net should also be considered, especially when covariates are highly correlated (Shen et al., 2016). Although providing daily information about tinnitus could increase user distress by repeatedly directing their attention to their tinnitus, previous work has suggested that using the TYT app does not harm users (Schlee et al., 2016). However, randomized control trials investigating the potential side-effects of recording intensive longitudinal data are still missing.

In summary, we show that tinnitus loudness and tinnitus distress are affected by both contemporaneous and lagged behavioral and emotional processes measured through EMA. Additionally, we showed that S-GIMME can distinguish users whose tinnitus distress and loudness are modulated by contemporaneous effects from those where those dynamics are modulated by both contemporaneous and lagged effects. Distinguishing these two subgroups could be therapeutical value, especially when aligned with just-in-time adaptive interventions to mitigate or prevent future peaks of tinnitus distress or increased loudness.

## Data Availability Statement

The raw data supporting the conclusions of this article will be made available upon request.

## Ethics Statement

The studies involving human participants were reviewed and approved by the Ethics Committee of the Faculty of Medicine of the University of Regensburg (Study approval number 15-101-0204). The patients/participants provided their written informed consent to participate in this study.

## Author Contributions

JS, WS, JB, and RP designed the study. JS, WS, and JB analyzed the data. JS, WS, PN, BL, and SM interpreted the results and conclusions. JS wrote the manuscript. All authors contributed to the article and approved the submitted version.

## Funding

This project has received funding from the European Union's Horizon 2020 Research and Innovation Programme, Grant Agreement Number 848261.

## Conflict of Interest

The authors declare that the research was conducted in the absence of any commercial or financial relationships that could be construed as a potential conflict of interest.

## Publisher's Note

All claims expressed in this article are solely those of the authors and do not necessarily represent those of their affiliated organizations, or those of the publisher, the editors and the reviewers. Any product that may be evaluated in this article, or claim that may be made by its manufacturer, is not guaranteed or endorsed by the publisher.
