# Supplementary material for: Daily Contributors of Tinnitus Loudness and Distress: An Ecological Momentary Assessment Study
Source: Front Neurosci. 2022 Jul 5;16:883665. doi: 10.3389/fnins.2022.883665 (PMC9294456; doi:10.3389/fnins.2022.883665)
Supplement: Supplementary file 1 [file Data_Sheet_1.docx]

**Daily Contributors of Tinnitus Loudness and Distress: An Ecological Momentary Assessment Study**

Supplemental material

**Methods**

**Auto- and Cross-Correlation**

Mathematically, the autocorrelation, r_k_, can be expressed as:

$$r_{k}=\frac{c_{k}}{c_{0}} Equation 1$$

where C_0_ represents the autocovariance of a variable at lag 0, and C_k_ represents the autocovariance for lag k, which can be mathematically described as:

where *k* represents a lag (each lag representing one day), *t*, represents the *t^th^* variable, and ** represents the mean of variable *x*. Similarly, cross-correlation can be described as the correlation between two variables at different lags and can be mathematically expressed as:

$$r_{k}^{x y}= \frac{g_{k}^{xy}}{\sqrt{\sigma_{x}\sigma_{y}}} Equation 3$$

where sigma represents the standard deviation of variables *x* and *y*, and g represents the cross-correlation function, which can be represented as:

$$g_{k}^{xy}=\frac{1}{n}\sum_{t=1}^{n-k} \left( y_{t} -\bar{y} \right)(x_{t+k} -\bar{x}) Equation 4$$

where *n* represents the sample size, *k* represents the lag, *t* represents the *t^th^* variable, and both ** and ** represents the mean of variables *x* and *y*.

**Elastic Net Regularization**

Elastic net combines two penalizing terms, *L1* and *L2*, and can be mathematically described as follows:

$$\sum_{i=1}^{n} \frac{{(y_{i}-x_{i}^{J})}^{2}}{2n}+\lambda(\frac{1-\alpha}{2}+\sum_{j=1}^{m} \hat{\beta}_{j}^{2}+\alpha\sum_{j=1}^{m} \left| \hat{\beta} \right|) Equation 5$$

Where *n* is the sample size, *i* represents the *i^th^* observation, j represents the *j^th^* predictor, $\hat{\beta}$ represents the estimated coefficients, lambda is the penalizing coefficient, and $\alpha$ is a tuning parameter: if set to 0, a ridge term is obtained, if set to 1, a lasso term is obtained. The first term represents the commonly used ordinal least squares (OLS) regression, the second represents an *L1* penalization (also known as ridge regression) and the third term represents an *L2* penalization (also known as lasso). If the lambda is estimated to 0, the output is a regular OLS regression. If not, the lambda must assume a positive value, in which the estimates from the regression are constrained. If a ridge penalization is used, the coefficients of regression are shrunk to values different than 0. Conversely, if a lasso regularization is used, the coefficients may be set to zero, which functions as a feature selection method. This powerful feature of the elastic net, to automatically select variables by setting some of them to 0, was used to build individualized models for each sequence of observations to predict both loudness (LO) and tinnitus distress (TD).

**Unified Structural Equation Modelling**

uSEM estimates contemporaneous and lagged relations (of order Q) with the following formula:

$$\eta_{t}=A\eta_{t}+ \sum_{q=1}^{Q} {\emptyset_{q}\eta}_{t-q}+\zeta_{t} Equation 6$$

Where $\eta_{t}$ the time series of length *t*, *A*, and $\emptyset_{q}$ contain the matrix of dimensions (*p, p*) of the contemporaneous and lagged (at lag *q*) relations, and $\zeta_{t}$ represents the residuals at time *t*.


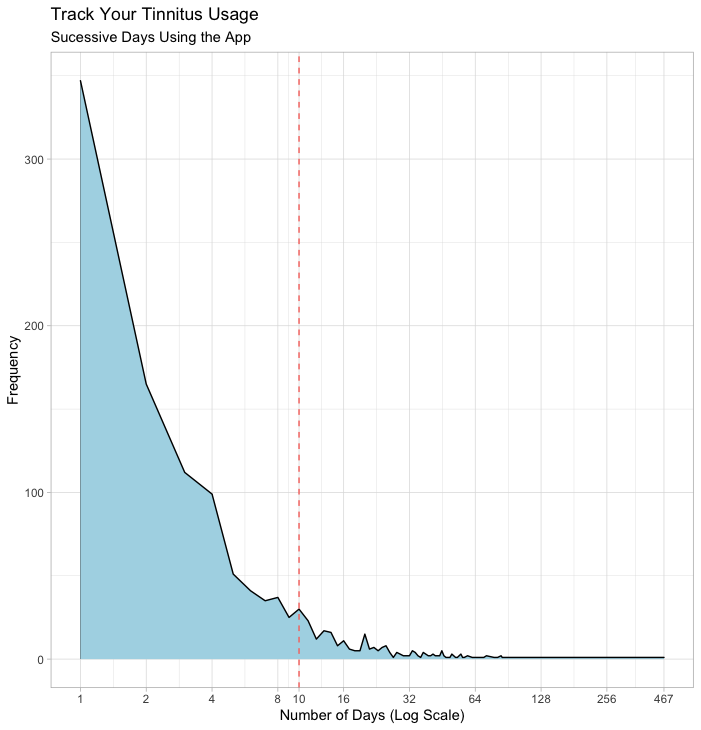


Sup. Figure 1 Overview of data. The x-axis shows the length of consecutive days in a logarithm scale ranging from one to 467 days, and the y-axis shows how frequent that sequence of consecutive days was observed. The red vertical dashed line shows the cut-off point of at least 10 days of consecutive observations that was used as inclusion criteria.

| User | chisq | df | npar | pvalue | rmsea | srmr | nnfi | cfi | bic | aic | logl |
| --- | --- | --- | --- | --- | --- | --- | --- | --- | --- | --- | --- |
| 1 | 63.96 | 45 | 59 | 0.03 | 0.05 | 0.06 | 0.97 | 0.98 | 5431.34 | 5244.96 | -2563.48 |
| 2 | 54.95 | 45 | 59 | 0.15 | 0.06 | 0.08 | 0.95 | 0.97 | 2040.61 | 1917.05 | -899.52 |
| 3 | 68.46 | 42 | 62 | 0.01 | 0.09 | 0.05 | 0.94 | 0.97 | 2480.38 | 2330.42 | -1103.21 |
| 4 | 55.06 | 41 | 63 | 0.07 | 0.05 | 0.05 | 0.93 | 0.96 | 5579.62 | 5388.3 | -2631.15 |
| 5 | 42.57 | 39 | 65 | 0.32 | 0.04 | 0.07 | 0.96 | 0.98 | 2386.66 | 2246.33 | -1058.17 |
| 6 | 68.88 | 45 | 59 | 0.01 | 0.06 | 0.07 | 0.96 | 0.98 | 3912.58 | 3744.77 | -1813.39 |
| 7 | 57.7 | 44 | 60 | 0.08 | 0.06 | 0.06 | 0.96 | 0.98 | 2518.83 | 2375.91 | -1127.96 |
| 8 | 72.41 | 45 | 59 | 0.01 | 0.07 | 0.06 | 0.96 | 0.97 | 4008.64 | 3839.46 | -1860.73 |
| 9 | 84.04 | 48 | 56 | 0 | 0.05 | 0.03 | 0.94 | 0.97 | 11988.77 | 11773.53 | -5830.77 |
| 10 | 47.22 | 42 | 62 | 0.27 | 0.04 | 0.07 | 0.97 | 0.98 | 3055.64 | 2904.93 | -1390.46 |
| 11 | 50.08 | 43 | 61 | 0.21 | 0.04 | 0.06 | 0.96 | 0.98 | 3033.89 | 2884.17 | -1381.09 |
| 12 | 56.69 | 40 | 64 | 0.04 | 0.05 | 0.05 | 0.96 | 0.98 | 5811.48 | 5610.79 | -2741.4 |
| 13 | 53.46 | 43 | 61 | 0.13 | 0.05 | 0.07 | 0.95 | 0.97 | 3018.87 | 2869.16 | -1373.58 |
| 14 | 53.33 | 46 | 58 | 0.21 | 0.05 | 0.07 | 0.95 | 0.97 | 2434.45 | 2306.58 | -1095.29 |
| 15 | 47.65 | 43 | 61 | 0.29 | 0.04 | 0.07 | 0.98 | 0.99 | 2010.34 | 1881.58 | -879.79 |
| 16 | 58.28 | 46 | 58 | 0.11 | 0.06 | 0.07 | 0.97 | 0.98 | 2215.59 | 2084.36 | -984.18 |
| 17 | 67.7 | 40 | 64 | 0 | 0.04 | 0.04 | 0.96 | 0.98 | 12272.59 | 12023.71 | -5947.85 |
| 18 | 66.34 | 42 | 62 | 0.01 | 0.08 | 0.06 | 0.95 | 0.97 | 2501.84 | 2348.24 | -1112.12 |
| 19 | 50.08 | 42 | 62 | 0.18 | 0.04 | 0.06 | 0.92 | 0.96 | 3919.21 | 3754.66 | -1815.33 |
| 20 | 53.33 | 44 | 60 | 0.16 | 0.06 | 0.08 | 0.96 | 0.98 | 2299.09 | 2166.81 | -1023.41 |
| 21 | 62.3 | 42 | 62 | 0.02 | 0.08 | 0.05 | 0.96 | 0.98 | 2209.39 | 2064.07 | -970.04 |
| 22 | 45.81 | 40 | 64 | 0.24 | 0.05 | 0.07 | 0.96 | 0.98 | 2169.26 | 2034.17 | -953.08 |
| 23 | 75.95 | 43 | 61 | 0 | 0.06 | 0.08 | 0.95 | 0.97 | 6906.86 | 6701.53 | -3289.77 |
| 24 | 48.92 | 43 | 61 | 0.25 | 0.04 | 0.06 | 0.97 | 0.98 | 2931.34 | 2783.79 | -1330.9 |
| 25 | 50.72 | 40 | 64 | 0.12 | 0.05 | 0.06 | 0.96 | 0.98 | 3016.32 | 2855.63 | -1363.81 |
| 26 | 55.39 | 45 | 59 | 0.14 | 0.06 | 0.08 | 0.95 | 0.97 | 2323.8 | 2191.99 | -1036.99 |
| 27 | 51.34 | 44 | 60 | 0.21 | 0.05 | 0.07 | 0.96 | 0.98 | 2190.08 | 2061.5 | -970.75 |
| 28 | 125.64 | 43 | 61 | 0 | 0.09 | 0.05 | 0.94 | 0.97 | 6447.8 | 6235.73 | -3056.87 |
| 29 | 62.99 | 44 | 60 | 0.03 | 0.07 | 0.05 | 0.97 | 0.98 | 2172.3 | 2030.13 | -955.06 |
| 30 | 190.92 | 45 | 59 | 0 | 0.18 | 0.04 | 0.93 | 0.96 | 393.07 | 242.39 | -62.2 |
| 31 | 56.17 | 43 | 61 | 0.09 | 0.05 | 0.05 | 0.96 | 0.98 | 4111.59 | 3941.04 | -1909.52 |
| 32 | 67.18 | 46 | 58 | 0.02 | 0.08 | 0.09 | 0.96 | 0.97 | 1794.71 | 1668.6 | -776.3 |

Sup. Table 1 Individual model fits from GIMME. All models converged normally. Chisq = Chi-squared, df = degrees of freedom, npar = number of parameters, rmsea = Root Mean Square Error of Approximation, srmr = Standardized Root Mean Square Residual, aic = Akaike information criterion, bic = Bayesian information criterion, logl = loglikelihood.
